# Conceptualizing impact in community-based participatory action research to engage communities in end-of-life issues

**DOI:** 10.1177/26323524221095107

**Published:** 2022-05-09

**Authors:** Max Kleijberg, Rebecca Hilton, Beth Maina Ahlberg, Carol Tishelman

**Affiliations:** Department of Neurobiology, Care Sciences and Society, Karolinska Institutet, Alfred Nobels Allé 23, Flemingsberg, 14183 Stockholm, Sweden; Research Centre, Stockholm University of the Arts, Stockholm, Sweden; Skaraborg Institute for Research and Development, Skövde, Sweden; Department of Sociology, Uppsala University, Uppsala, Sweden; Department of Learning, Informatics, Management and Ethics, Karolinska Institutet, Stockholm, Sweden; Stockholm Health Care Services (SLSO), Region Stockholm, Stockholm, Sweden

**Keywords:** community-based, end-of-life, health-promoting palliative care, impact, participatory action research

## Abstract

**Background:**

A health promotion approach to end-of-life (EoL) care is gaining traction internationally. However, there is a lack of evaluations of the impact of this approach, particularly regarding community-based initiatives. Conceptualizations of impact in participatory action research (PAR) may contribute to understanding ways in which impact can be investigated in community-based health promotion approaches to EoL issues. We aim to investigate impact and the process of impact development in our community-based PAR project, Studio DöBra, a Swedish health promotion initiative to engage communities in EoL issues.

**Methods:**

We do this through a qualitative framework analysis expanding on Banks *et al.*’s theory of co-impact in PAR, based on longitudinal empirical data of Studio DöBra. Studio DöBra was developed in partnership with a range of community organizations and engaged children (9 years old) and older adults (most 80+) with topics related to dying, death, and loss through arts activities. The analyzed empirical data reflect the perspectives of community-partners and academic partners from interviews and meetings spanning 4.5 years.

**Findings:**

We present a model of impact development consisting of impact on individual and group development, action-oriented impact, and strategy-oriented impact; ways they relate to and evolve from one another; and how they may be affected by contextual influences.

**Conclusion:**

Besides contributing to conceptualizations of impact in PAR, findings contribute a community perspective to the limited literature investigating the impact of health promotion initiatives related to EoL issues.

## Background

A health promotion approach to palliative care is gaining traction internationally in response to issues related to aging populations and a professionalization of end-of-life (EoL) care.^1–3^ Based on the notion that the EoL primarily is a social issue that concerns everyone, health promotion approaches to palliative care often work with communities to increase engagement in EoL issues to enhance a sense of control and support for those who are dying, grieving, or are providing care.^2,4–6^ Although the relevance of these initiatives is increasingly recognized, researchers in this field have pointed to the lack of evaluations of the impact of this approach.^3,7,8^ Furthermore, evaluations of community-based initiatives are particularly lacking.^7–10^

Participatory action research (PAR) may be applied to both conduct and study community-based health promotion approaches to EoL issues. In PAR, academic and community-partners collaborate to bring about meaningful change for those involved and together develop knowledge for practice.^11,12^ However, it is often challenging to understand the processes through which such change occurs.^13^ This is in part due to the emergent and context-specific nature of PAR.^14^ In addition, while PAR often has explicit change goals, unplanned changes also occur.^15^ Efforts to conceptualize impact in PAR are one important way to better understand how change occurs through PAR^13–17^ and may contribute to understanding ways in which impact can be investigated in relation to community-based health promotion approaches to EoL issues.

Based on a PAR project about debt in low-income households and broader PAR experiences, Banks *et al.*^14^ conceptualized three types of ‘co-impact’ which form the basis of the analysis presented in this article. Banks *et al.*^14^ call the first type ‘Participatory impact’, which is process-based and refers to ‘changes in the thinking, emotions and practice of researchers and core-partner organizations, which happen as a result of their involvement in conducting PAR’. The second type is ‘Collaborative impact’, which is findings-based and refers to ‘the take-up and use of the findings of collaborative research by individuals and organizations to change practice and policy, and influence attitudes and culture’.^14^ The third type is ‘Collective impact’, which involves ‘a deliberate strategy on the part of the research partners (and sometimes others) to achieve a specific, targeted change in practice and/or policy based on issues highlighted via the research’.^14^ This third definition appears somewhat contradictory, as Banks *et al.* highlight that PAR processes are emergent, although emergent forms of impact are not included in their definition.

In this article, we build further on the work of Banks *et al.*, as we aim to investigate impact and the process of impact development in our community-based PAR project, Studio DöBra. In this manner, we seek to contribute to conceptualizations of impact in community-based health promotion approaches to EoL issues.

### Background and context of Studio DöBra

Studio DöBra (*DöBra* is a Swedish pun literally meaning dying well, but figuratively meaning ‘awesome’) is a community-based initiative in which children (9 years old) and older adults (most 80+) engage with topics related to dying, death, and loss through arts activities. Goals include supporting community engagement in issues related to dying, death, and loss and creating opportunities for interaction between children and older adults.^18–20^ Studio DöBra was developed in partnership with community organizations with the DöBra research program as the academic partner. Two Studio DöBra iterations were developed in different Swedish cities (2016, 2017–2018), each involving eight children and eight older adults in a series of five arts workshops. Partners from both iterations thereafter developed the Studio DöBra Toolbox together, a resource to document and disseminate lessons learned.

The development of Studio DöBra was motivated by the Swedish context with an aging population but few intergenerational meeting places, which can contribute to age segregation, ageism, and loneliness among older adults.^21,22^ Another motivation was relatively low public engagement with end-of-life (EoL) issues in Sweden compared with many European countries.^23–25^ Studio DöBra was therefore conceptualized as a health promotion approach to strengthen community engagement with EoL issues. Through involving organizations not usually engaged with such issues and inviting children and older adults, who were not in need of palliative care, Studio DöBra promotes early and intergenerational engagement with such issues.

#### The Studio DöBra process

Studio DöBra was informed by principles of what is generally termed community-based participatory research.^11^ Although this term is well established, we use the term community-based participatory action research (CBPAR) because both action leading to change and the development of new knowledge through practice have been central to our approach.^26,27^

The relatively low level of community engagement with EoL issues in the Swedish context, combined with the academic partner as initiator, led us to anticipate difficulties in establishing community partnerships for the first iteration in 2016. We were, however, met with immediate and positive response from prospective partners.^19^ Representatives from a children’s library, an artistic organization for children, an activity center for older adults, and an after-school center in a multi-ethnic urban area outside a large city, plus M.K. as academic partner, formed a project group to develop the first Studio DöBra iteration. They invited children and older adults as voluntary participants. After concluding the first iteration, partners maintained contact formally (e.g. analysis, interviews, presentations) and less formally (e.g. social media, email and in the community).

In 2017, the municipal organization for culture in a mid-sized city in another part of Sweden learned about Studio DöBra through word of mouth. They then invited M.K. to help them develop a second iteration in this city. They formed a project group including representatives from the municipal organizations for culture and elder care, an after-school center, and M.K. While M.K. had a leading role as initiator in the first project group,^19^ in the second group representatives from the municipal organization for culture took the lead. M.K. contributed with experiences and lessons learned from the first iteration.

After the end of the second Studio DöBra workshop series, partners from both iterations began to consider ways to document and share learnings with those interested in developing similar initiatives as well as with the general public. In addition, community-partners expressed the need for material to help support them when reporting to management and politicians. Moreover, as the project groups had never met, there was interest from both groups to exchange experiences and learning from each other.

**Figure 1.**
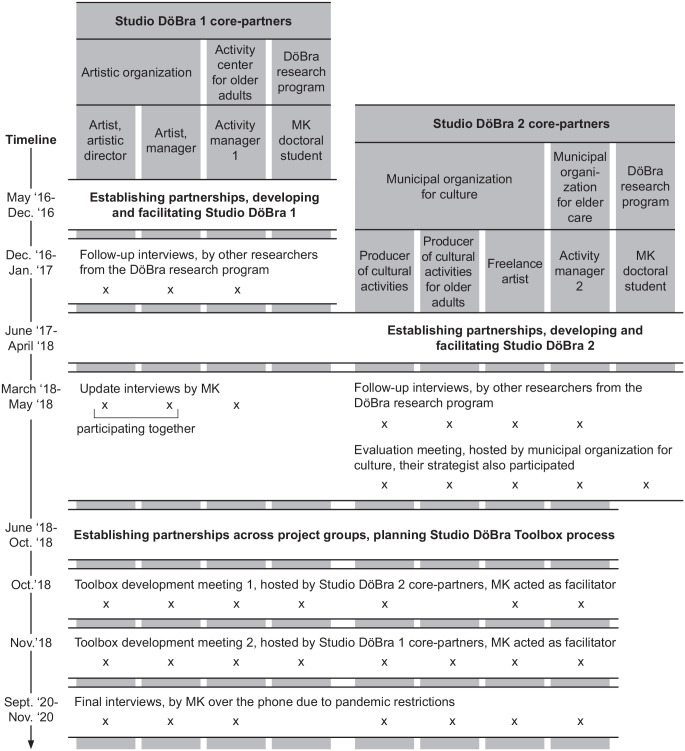
Core-partners from each Studio DöBra iteration, and overview of the longitudinal data underlying this article. *Note.* MK was part of both Studio DöBra 1 and 2. Core-partners from both iterations participated in developing the toolbox, MK is therefore only included there once (from June ‘18).

These ideas led partners from both cities to begin to develop the Studio DöBra Toolbox in 2018. Due to a lack of professional mandate, not all community-partners participated in this process. Those who did, including the academic partner M.K., are referred to as core-partners as listed in Figure 1. The core-partners from each project group hosted a full-day Toolbox development meeting in their city, after which further collaboration took place online. M.K. facilitated these meetings, as he was the only person who participated in both groups. The Toolbox contains practical tips for developing similar initiatives and includes examples of Studio DöBra arts activities. It is available both in print and digitally, free of cost on the DöBra website.

While our previous research investigated experiences and impact of participating in Studio DöBra from the perspectives of children and older adults,^18^ we focus here on the perspective of core-partners. Two were men and six were women. At project commencement, they had an age range of 28–48 years.

## Methods

### Ethical considerations

Throughout the CBPAR process, ethical considerations were guided by the notion of reciprocity in academic-community partnerships.^28^ As M.K. had a central role in Studio DöBra as initiator and academic partner, power dynamics had to be navigated to develop and maintain reciprocal partnerships. Prior Studio DöBra research investigating this found that collective reflective practice helped to ensure that partnerships and participation were of mutual benefit for both academic and community-partners.^19,29^ Ways of dealing with confidentiality and acknowledgment of partnerships throughout this CBPAR were agreed upon from the outset and were continually discussed and updated as new situations arose.^30^ In consultation with core-partners, we here omitted sensitive personal information, specific locations, and names of community-partners and their organizations.

### Data generation

A variety of qualitative data was generated with core-partners throughout the CBPAR process spanning a period of about 4.5 years, allowing us to investigate change over time. Data sources are shown in Figure 1. All interviews and the evaluation meeting after Studio DöBra 2 were audio-recorded and professionally transcribed. Interviews were conversational, focusing on the meaning and implications of participation on both individual and organizational levels, reflections on partnerships, and future plans. Interviews conducted by M.K. also included reflecting together on lessons learned, as did the evaluation meeting, which included discussing ways in which lessons learned could be shared with others. The two full-day Toolbox development meetings were audio-recorded with key sections transcribed by M.K. The first meeting involved mapping lessons learned; during this process, partners reflected together on the meaning and implications of participation on individual and organizational levels. During the second meeting, partners worked with the mapping of lessons learned to form a first draft of the Toolbox. Documentation of M.K.’s reflective practice is also included as data and entailed written personal reflections and notes from reflective conversations with co-authors.

### Analysis

The analysis process was inspired by a framework analysis approach, which involves the conceptualization of a thematic framework based on a priori issues from, for example, literature and early ideas about the data.^31^ This provided guidance for our use of existing theories to support empirical investigation and further development of these theories. Analysis was led by M.K. in a process in which authors repeatedly reflected on and discussed preliminary findings. Core-partners contributed feedback in a group meeting as described below. As M.K.’s own reflections were part of the data, co-authors supported M.K. in critically considering his own reflections and interpretations.

**Figure 2.**
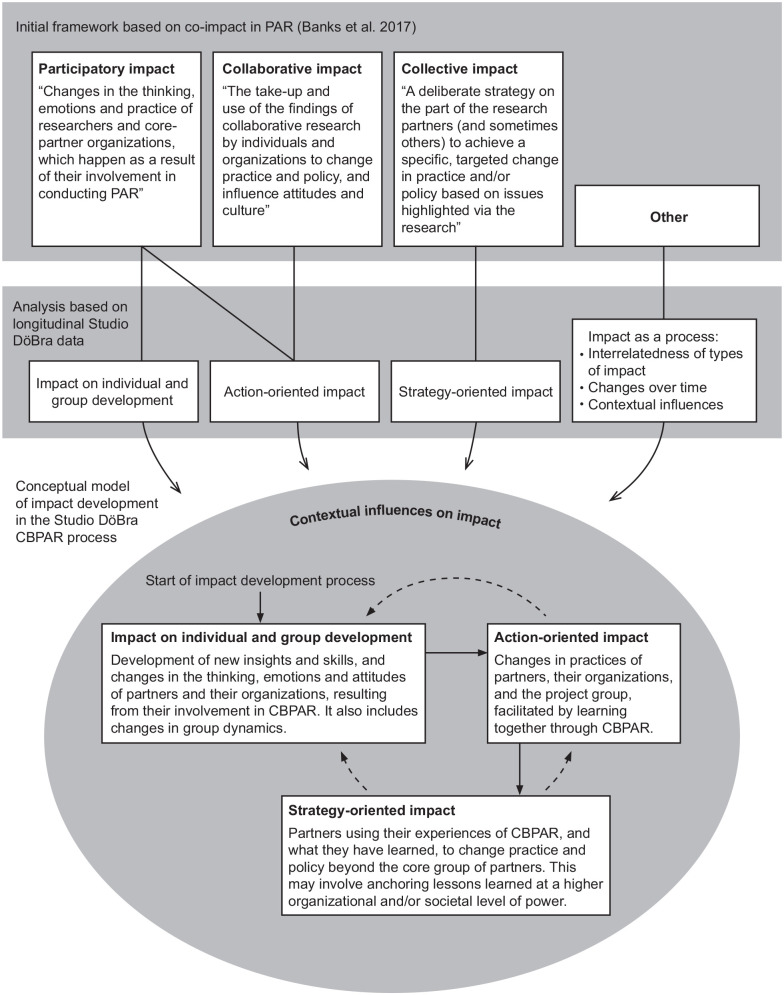
Schematic visualization of the analysis process and our conceptual model of impact development. *Note.* In the conceptual model, arrows represent impact as a process. Solid arrows show the order in which types of impact were developed over time. Dotted lines show the feedback loop which made interrelatedness reciprocal.

M.K. began the analysis by listening to audio-recordings and reading transcripts, thereafter creating one case for each core-partner by chronologically compiling all data related to that person, using NVivo software. Banks *et al*.’s^14^ conceptualization of impact, described above, formed the basis for the initial coding framework created in NVivo (see Figure 2). An additional code (‘other’) was included for relevant data that did not fit into the existing codes. One challenge was to adapt the framework analysis approach to incorporate our longitudinal qualitative data.^32^ M.K. did this by memo-writing during coding, focusing on connections between codes and changes over time, and through reflective discussions with co-authors.^32^

As M.K. began to code data, it became apparent that Banks *et al.*’s types of co-impact could overlap, something which Banks *et al.* also highlighted.^14^ In addition, we found that the development of different types of impact was interrelated in our longitudinal data, an aspect not accounted for in Banks *et al.*’s conceptualization which treats impact only as the outcome of a process. This led us to consider the *process* of impact development as of interest in itself, that is, impact as a process.

Based on the above, we therefore further developed Banks *et al*.’s conceptualization in an iterative process. During the analysis of data from each core-partner, we adjusted and clarified definitions to better distinguish types of impact, considering factors in relation to impact as a process (see Figure 2). Throughout the analysis, we consulted other PAR literature^27,33–35^ to gain perspective on both Banks *et al.*’s theory and our evolving findings and help us fine-tune our definitions of types of impact. Based on this analysis, we were able to conceptualize a model of impact development in Studio DöBra based on impact both as the outcomes of a process and as a process itself, as illustrated in Figure 2.

Community-partners had early on decided not to participate in the analysis phase described above but wanted to give feedback in a later stage. Thus, M.K. held a virtual meeting (due to COVID-19 constraints) with community-partners to reflect on and discuss tentatively formulated findings. The partners confirmed our interpretations, reflecting on ways the model helped them to see links between their own impact processes and outcomes, and giving them new theoretical perspectives on phenomena they recognized from experience.

## Findings

Based on the analysis of longitudinal data from core-partners’ perspectives throughout the Studio DöBra CBPAR process, we build further on Banks *et al*.’s^14^ theory of co-impact and present here a model of impact development which describes impact both as a process and as an outcome (Figure 2). We distinguish three types of impact: impact on individual and group development, action-oriented impact, and strategy-oriented impact. These bear similarities to Banks *et al.*’s original categorization but differ in ways outlined below.

In presenting findings, we first define the types of impact in general terms. We then discuss impact as a process, providing examples of the types of impact in Studio DöBra and how they were developed. Here, we first focus on processes leading to impact on individual and group development and action-oriented impact, followed by a section on developing strategy-oriented impact and concluding with dis/continued impact development. We draw from PAR literature used in analysis and exemplify findings with empirical data, referring to data sources as shown in Figure 1.

### Three types of impact

#### Impact on individual and group development

Based on our analysis, we define impact on individual and group development as the development of new insights and skills, and changes in the thinking, emotions, and attitudes of partners and their organizations, resulting from their involvement in CBPAR. It also includes changes in group dynamics, such as establishing new social connections. Figure 2 illustrates that our construction of this impact type originates from Banks *et al.*’s^14^ participatory impact. However, whereas Banks *et al.*’s^14^ participatory impact includes changes in both partners’ mindsets and practices, we focus on mindset changes and include practice-related changes in what we call action-oriented impact. Another difference is that we include changes in group dynamics as we found that individual and group development went hand-in-hand in Studio DöBra, as illustrated by the producer of cultural activities, in the follow-up interview:

*Something I found very interesting and important in the project, in the first phase, was that we quickly began to talk about … or ransacked ourselves a bit, and tried to think about our own personal experiences of the issues that the project deals with, so death and loss, and people living close to death […] So we talked quite a lot about that, and that was something which we tried to bring with us into the project.*

Thus, reflecting together on personal experiences affected partners not only individually but also as a group, informing their practice in Studio DöBra. This also illustrates our finding that individual and group development was a prerequisite for action-oriented impact, which we elaborate below. Impact on personal and group development in Studio DöBra includes, for example, meaningful connections and interactions among partners and participants (e.g. developing friendships and partnerships) and developing interest, confidence, and skills to support intergenerational interactions, support engagement with EoL issues, and use arts activities to facilitate this.

#### Action-oriented impact

Based on our analysis we define action-oriented impact as changes in practices of partners, their organizations, and the project group, facilitated by learning together through CBPAR. In our data, we found that action-oriented impact first occurred when partners applied their new/changed insights, skills, and attitudes (i.e. impact on individual and group development) to their own practices and policies.

Figure 2 illustrates that action-oriented impact originated from both Banks *et al.*’s participatory impact as noted above and collaborative impact which revolves around the uptake of collaborative research findings. However, during analysis, we began to question what is defined as a research finding in CBPAR, as this approach aims to democratize knowledge development to include multiple ways of knowing, rather than privileging an academic epistemology.^27,33,34,36^ We found, for example, that Studio DöBra community-partners had already integrated experiential and practical knowledge into their practices, while academic analysis was still ongoing, as illustrated by this excerpt from the update interview with Studio DöBra 1 artists:

Artist, artistic director: *What I find interesting now, is that you are writing* (referring to M.K.’s doctoral thesis) *about what we’ve done, while we* (referring to the artists) *talk about that knowledge and … what we’re going to do tomorrow. We’re already into a future vision, and that’s a kind of… a quickness. That’s quite interesting* [that] *new knowledge emerges through an interaction and how this knowledge is used in different ways. In your case, you go through all the documentation very carefully…*

M.K.: *Exactly, yes.*

Artist, artistic director: *And you come with* [results], *while we’ve already taken it and applied it several times and developed it further… it’s a part of our concept for the future.*

Thus, in Studio DöBra, knowledge developed through the interaction of different ways of knowing among academic and community-partners as well as collaborative action and reflection. We therefore use the phrase ‘learning together’ in our definition of action-oriented impact to encompass a multiplicity of knowing.^27,35^

We found that action-oriented impact could involve changes in practices both within and outside Studio DöBra. Impact within Studio DöBra included, for example, partners continuously applying lessons learned in following workshops, M.K. transferring lessons learned from Studio DöBra 1 into Studio DöBra 2, partners applying lessons learned in developing the Toolbox, and a shift from academic partner M.K. as initiator to initiatives driven by community-partners, as illustrated by the Toolbox. Impact outside Studio DöBra included, for example, partners creating spaces for intergenerational interaction and engagement with EoL issues in their own professional practices and social networks.

#### Strategy-oriented impact

Based on our analysis, strategy-oriented impact relates to partners using their experiences of CBPAR, and what they have learned, to change practice and policy beyond the core group of partners, for example, by involving new partners or organizations and creating spinoff projects. This may involve anchoring lessons learned at a higher organizational and societal level of power. In Studio DöBra, the Toolbox was a spinoff project that partners used in knowledge exchange sessions to involve others in EoL issues. This was done through workshops and presentations with care staff and the general public. The Toolbox was also used to anchor lessons learned with managers and politicians. Additional spinoff projects developed as community-partners used the Toolbox in new settings, for example, dementia and home care. In addition, the second Studio DöBra iteration can be seen as a form of strategy-oriented impact as it involved new partners.

We based strategy-oriented impact on Banks *et al.*’s^14^ collective impact, as shown in Figure 2, as we adopted their ideas about partners strategically aiming to create change. In contrast to action-oriented impact, strategy-oriented impact is about change occurring beyond core-partners. In analyzing our longitudinal data, we found that partners began to formulate ideas for strategy-oriented impact based on individual and group development and action-oriented impact. We therefore concluded that in this project, impact on individual and group development and action-oriented impact were prerequisites for strategy-oriented impact.

It should be noted that in our definition, we exclude Banks *et al.*’s^14^ criterion for collective impact that agendas for change are ‘shared by all parties’. This is based on our finding that in collective processes, partners could have both shared and separate goals for strategic change. For example, the Toolbox development was a collective, shared process, while partners’ goals for its strategy-oriented impact differed, for example, some wanted to anchor lessons learned with management and local politicians, while others wanted to use it to create spaces for engaging with EoL issues in other contexts, as illustrated with data excerpts below.

### Impact as a process

#### Processes leading to impact on individual and group development and action-oriented impact

Processes leading to impact on individual and group development and action-oriented impact are inextricably related as they result from iterative and interactive action and reflection in our CBPAR process, exemplified by activity manager 2’s reflection in a follow-up interview: ‘You have to try things and draw conclusions and then try again. That’s interesting’.

We therefore describe the relationship between the processes of developing impact on individual and group development and action-oriented impact as reciprocal. However, as noted above, we found that this relationship began with individual and group development in both Studio DöBra iterations. One example is that partners described reflecting together on EoL issues and aging (i.e. impact on individual and group development) as a basis for informing practice (i.e. action-oriented impact), exemplified by the first quote in the ‘Findings’ section.

One example of individual and group development leading to action-oriented impact beyond Studio DöBra is that partners commonly described gaining confidence and skills in broaching EoL topics and other sensitive issues and then linked to creating space for conversations about these issues in both their social networks and professional practices as illustrated by the freelance artist in the final interview:

*Well, it’s mostly about talking about it, whatever it is we’re talking about, to dare to and to establish a framework maybe. So as a pedagogue or facilitator or whatever, you make it easy for participants to follow the theme. […] And to give it space, and not to dismiss it or joke it away. But to let people talk about it, and maybe support and unravel it.*

An example of the reciprocal relationship between these two types of impact is that the artistic organization from the first iteration began to include older adults as a target group, although prior to this they had worked exclusively with children. This in turn led to impact on individual and group development, as the artists began to develop personal relationships, particularly with two older participants, Stig and Gunnar (pseudonyms), who became quite involved in the organization, as exemplified by the following excerpt from the update interview in which both artists participated:

Artistic director: *Stig contacted me* [right before the summer], […] *he was creative himself* […] *and he wanted to show his tools and machines to me* […] *and he wanted me to have them, and I didn’t understand back then, but you* (addresses artist manager) *understood that something was going on.* […] [We met] *three or four days before* [vacation]. *We had lunch and looked at* [his] *workshop, and he was pretty perky. And then we separated, and I went* [on vacation] *and the phone rang* […] *and it was Stig. You should know that Stig and Gunnar call us rather often sometimes, even if it’s not important* (laughs) […] *so I didn’t take the call, you know, you’re on vacation. And then a few days later, Gunnar called, pretty undramatically he just said ‘Stig passed away two days ago, in the hospital, and I just wanted to let you know’. And I was flabbergasted. Nothing like this had ever happened to me. But* [Gunnar] *was calm in his tone and so damn stable.*

Artist manager: *Always.*

Artistic director: *So, I also became stable. And then nothing happened. I’d seen the workshop and he wanted to give those things to me, but I hadn’t been in contact and I don’t want to have… or have the need to have them.* […] *It got me thinking, yes of course, that’s what I’ll do too, right? You have a lot of tools and stuff you’ve collected. It’s a beautiful gesture to give that to another creative person who still… or to a young person who needs those things. So that’s the story about Stig.*

The artists expressed ways in which the children who had met Stig reacted to his death by posting about it on social media and lighting candles at the local graveyard. The artist manager said, ‘It became so clear that, wow… this is also a part of [our organization]. So that was very beautiful’.

The story about Stig illustrates the reciprocal relationship between action-oriented impact and impact on individual and group development. It demonstrates how the evolving practice of the artistic organization impacted social relationships and in turn impacted how the artists viewed their organization. In addition, although the artists focused on continuing to create spaces for intergenerational interaction beyond Studio DöBra, they began to recognize that in doing so they had also created spaces for dealing with experiences of death and loss.

A facilitating contextual influence was the structure of the artistic organization which allowed the artists themselves to make decisions about expanding the target group. However, a contextual influence hindering sustainability was the organization’s struggle to secure ongoing funding at the time of the update interview. The structure of the municipal organizations in the second iteration presented other examples of contextual influences hindering or facilitating sustainable impact. The producer of cultural activities for older adults and activity manager 2 discussed continuing to work closely together as a result of Studio DöBra, facilitated by a pre-existing agreement between the municipal organizations for culture and elder care. While this agreement is intended to increase interaction and collaboration, the producer of culture for older adults reflected on its challenges in the final interview:

[The collaboration] *is based on the relationships you develop with each other. And you can say what you want, that* [the agreement] *shouldn’t be bound to individuals and so on, but that’s how it is.* […] *We’ve seen in other municipalities, where the collaboration doesn’t work* […] *that the agreement dies quickly* […] *then the funding we have also disappears.* […] *So, it’s very important to have a good and functioning collaboration, really.*

Thus, dependency on individual commitment and relationships can stimulate continued impact but also risk its sustainability.

#### Developing strategy-oriented impact

Through our analysis of longitudinal data, we found that partners began engaging in efforts to develop strategy-oriented impact after they developed and applied new/changed interest, skills, and attitudes in their own practices. This led, for example, to the Toolbox initiative. We found that partners with various agendas and motivations could simultaneously work on a common goal for strategy-oriented impact, without compromising specific goals. This is exemplified by a discussion during Toolbox development meeting 1, about the form the Toolbox should take, summarized and illustrated with data excerpts below.

M.K. assumed that partners would have limited time and funding to work on Toolbox development, so he proposed the Toolbox take the form of a draft of a written document. It became clear, however, that community-partners had more ambitious expectations. The freelance artist, for example, had ideas about developing digital solutions and books, while activity manager 1 had ideas for spinoff projects to create spaces for engagement with EoL issues in new contexts. She explained that she needed physical material to trigger conversations, saying, ‘I think that it’s been so hard, how do I broach the topic? But if I have material to present, then it becomes much easier for me’.

In line with these ideas, Studio DöBra 1 artists explained,

Artist, manager: *We always work with the physical, if you see how it facilitates* [the conversation] *if you have something to hold in your hands.* […] *And that’s how we’ve worked* [in Studio DöBra].

[…]

Artist, artistic director: *If it would be a product from just* [our organization], *which it isn’t because it’s a collaboration, then it would definitely be a box* […] *with material* […] *we strongly believe in the physical.* […] *If you have a physical object* […] *it’s much easier to communicate* [the project to others]. […] *That’s how we work.*

Rather than compromising on individual goals, partners decided to develop a digital toolbox together, also available in print. Studio DöBra 1 artists also developed a physical toolbox, consistent with their ways of working and specific to the needs of activity manager 1. Core-partners acquired the necessary resources to make their ambitions feasible.

We found a reciprocal relationship between the processes of developing strategy-oriented impact and the other two types of impact, which can be exemplified by activity manager 1’s process throughout the CBPAR. Through the original project, and in developing and using the digital and physical toolboxes, she became increasingly confident in creating spaces for engagement with EoL issues in various work contexts. She now facilitates spinoff projects in an elder care center working with dementia care staff. In addition, her manager invited her to facilitate another project involving home aides for individuals with specialized palliative care needs. Thus, for activity manager 1, the process of creating strategy-oriented impact also led to both individual development and action-oriented impact.

We also found that partners made efforts to anchor lessons learned by involving politicians and managers on higher power levels, that is, with agency to affect different types of change. This met with varying degrees of success. As noted above, activity manager 1 acquired funding and support from her manager, indicating anchoring at a higher level of power. On the other hand, while Studio DöBra 2 community-partners received positive feedback from management and politicians, activity manager 2 reflected in the final interview on hindrances related to contextual influences:

*I’ve thought a lot about what* [decision makers] *rely on when they make decisions, and what type of research results* […] *It’s like that in society in general* […] *that people want to see statistics and percentages* […] *And that people don’t consider quality more.* […] *That’s why it’s very good to be able to rely on… well that I have a researcher who backs this up* […] [I] *notice that when* [I] *mention* [names the academic medical faculty] *people take it more seriously.*

Thus, in Studio DöBra, although academic and community-partners worked together to develop new knowledge in line with democratizing knowledge development in participatory research,^27^ the prevailing societal notion that valid knowledge is developed in academic institutions^36^ limited community-partners’ ability to independently anchor lessons learned on higher power levels. Community-partners reflected on using their collaboration with a respected academic medical faculty to gain legitimacy with external stakeholders and decision makers. This illustrates one way in which community-academic research partnerships may support community-partners to deal with existing hierarchies, potentially leading to empowerment in developing strategy-oriented impact.

#### Dis/continued impact development

The illustration of our model in Figure 2 implies that, in theory, once the process of impact development started in Studio DöBra, it continued indefinitely. We found, however, that in practice, continued impact was dependent on contextual factors, including support from decision makers, (dis)continuity of management, possibilities within job descriptions, availability of resources, dependency on personal engagement and relationships, and, at present, limitations posed by the COVID-19 pandemic. All community-partners spoke about needing to adapt their ways of working since the pandemic, as it became impossible to meet in groups and across generations. This resulted in paused, postponed, canceled, and adapted projects and altered job descriptions.

Throughout the CBPAR process, community-partners reflected on continued contact with M.K. as academic partner as a factor supporting continued impact development. Activity manager 1 reflected in the update interview:

*We’ve had a really good collaboration, I think, and it’s fun to – for the first time – be part of a project that you get to continue.* […] *It’s not just a project here and now and then it ends, and then you don’t know anything. Here we’ve really been able to follow up* […] *and we still have this contact* […] *so it stays alive in a way.* […] *so it feels more rewarding as well.*

She returns to speak of this in the final interview:

*I see a continuation from* [Studio DöBra] *because I’ll do* [a spinoff project] *in my current job also* […] *So it doesn’t end because it becomes something else, just in another form, but anyway it continues. I think it’s fun and exciting that it lives on.*

In the final interview, the producer of cultural activities said that impact development had ceased for her because she had changed jobs. However, upon reflection, she recognized how her experiences from Studio DöBra might be applicable in her new job:

*But I hadn’t thought about that earlier, that connection, but now I can see it* (laughs).

This illustrates how the interviews themselves could stimulate continuing impact development.

## Discussion

In this article, we seek to contribute to conceptualizations of impact in community-based health promotion approaches to EoL issues. We do this through qualitative framework analysis, utilizing longitudinal empirical data from our CBPAR project Studio DöBra, to expand on Banks *et al*.’s^14^ theory of co-impact. We present a model of impact development, including three types of impact (i.e. impact on individual and group development, action-oriented impact, strategy-oriented impact) and ways in which they relate to and evolve from one another, and were affected by contextual influences. By approaching impact as both process and outcome, our model considers the evolving and emergent nature of PAR and community-based health promotion approaches to EoL issues.

We developed the model based on a health promotion initiative to engage communities in EoL issues, as well as on Banks *et al.*’s work on a PAR initiative about debt in low-income households, two very different contexts. We therefore believe that the model has potential for use in further contexts, and its development can continue by adapting and applying it to other PAR initiatives. In addition, our findings add to the limited body of literature investigating impact of health promotion initiatives to engage communities in EoL issues.^1,3,7^ This literature remains dominated by studies on initiatives linked to healthcare institutions.^3,7^ This article contributes to this literature with findings on impact from a community perspective.

The model connects with the various aspects of health promotion as described in the Ottawa Charter for Health Promotion.^37^ Impact on individual and group development relates to health promotion efforts to develop personal skills^37^ and may also be understood as a form of gaining death literacy.^38^ Action-oriented impact relates to strengthening community action in health promotion.^37^ Strategy-oriented impact relates to the Ottawa Charter’s systemic goals of building healthy public policy, creating supportive environments and reorienting health services.^37^ The Ottawa Charter points to prerequisites for health, for example, socioeconomic factors,^37^ which relate to contextual influences on impact development. While the Ottawa Charter describes various health promotion goals separately, our model creates explicit links between various forms of impact, which may facilitate the understanding and assessment of impact development through health promotion.

Governments and funding agencies increasingly demand that research leads to social change.^13,39–41^ PAR is meant to affect social change through co-producing knowledge and using this knowledge to inform practice in an iterative manner.^11,33^ However, the established ways of investigating and reporting research impact, as a linear transference of new knowledge directly from research to society, reveal limited applicability in the context of the evolving, unfolding, and interactive nature of PAR.^13,40^ Our model contributes to a broader understanding of impact, namely as both process and outcome, demonstrating that various types of impact evolve from one another over time and are subject to contextual influences. This implies that while it is important to report strategy-oriented impact, arguably the type of impact which governments and funding agencies are most interested in, it is equally important to account for impact on individual and group development and action-oriented impact as they may provide the basis for strategy-oriented impact, as in Studio DöBra. We therefore emphasize the importance of evaluating all three types of impact when assessing PAR.

Findings indicate that community-partners who traditionally did not engage with EoL issues began to do so through developing interest, confidence, and skills and subsequently applying them to practices and policies within and beyond their organizations. This can be understood as a participatory learning process leading to empowerment in relation to EoL issues.^42,43^ Therefore, our model of impact development may be relevant for investigating empowerment in community engagement with EoL issues.

One way to understand impact development as beginning with individual and group development is as a process of gaining ‘power to act’ to use Gaventa and Cornwall’s term.^33^ They argue that an understanding of power in PAR goes beyond a ‘“power over” relationship’, since ‘power to act’ and ‘power to act in concert with others’ are ‘fundamental to transformational social change’ (p. 467).^33^ Thus, through individual and group development, Studio DöBra partners gained ‘power to act’ and ‘power to act in concert with others’, which they then applied to change their own practices, leading in turn to strategy-oriented impact.

Findings indicated that efforts to achieve strategy-oriented impact included upstream endeavors to anchor lessons learned at higher levels of power. This had varied success due to contextual influences. Using Gaventa and Cornwall’s terms, these contextual influences can often be ascribed to ‘power over’ dynamics.^33^ In Studio DöBra, this relates to the power which management and politicians wield over community-partners, either facilitating or hindering anchoring lessons learned at higher levels of power. Another way to look at these upstream endeavors is through a social ecology lens, which situates power dynamics in participatory research within micro (individual values and attitudes), meso (inter-sectorial spaces), and macro (socio-political structures) levels, as proposed by Roura.^44^ As with Studio DöBra, PAR projects are often situated on grassroot, micro, and meso levels,^27^ but as Roura points out, support on the macro level is essential for anchoring lessons learned and thus achieving sustainable social change.^44^ Our findings indicate that systemic and cultural changes are needed for community-produced knowledge to be recognized as being equally valid as researcher-derived knowledge beyond the core-partner group on macro level.

Some issues should be considered when interpreting our findings. Data underlying this research are limited to two iterations of Studio DöBra in two different Swedish cities, each involving eight children and eight older adults. Furthermore, the model is developed solely from the perspectives of core-partners, and thus does not include perspectives from higher levels of organizational and societal power; this may curtail insight into strategy-oriented impact. The investigation of spinoff projects, and dissemination and use of different toolboxes may provide deeper understanding of strategy-oriented impact. In our previous research based on the perspectives of the participating older adults, children, and their parents, we found that older adults and children began to create spaces for EoL engagement in their social networks after participating in Studio DöBra, for example, family conversations at the dinner table.^18^ This can be seen as impact related to individual development and action-oriented impact, but more research is needed to further develop the model through perspectives from stakeholders beyond the core-partners. Ideally this would also include stakeholders at the macro level. The focus on the perspective of core-partners in Studio DöBra also limits the understanding of the full scope of contextual influences on impact development, such as socioeconomic and cultural factors enabling or hindering participation. Prior Studio DöBra research indicated that partners’ participation was based on and facilitated by a social and professional mandate to action.^19^ Perspectives from participating children and older adults are needed to understand contextual influences particular to their experience. Despite our awareness that models are inherently reductionist, we use one here to help tease out and further understand the complexity of impact development in CBPAR.

In summary, we contribute to conceptualizations of impact in community-based health promotion approaches to EoL issues by considering impact as both process and outcome, as different types of impact relate to and evolve from one another. Our findings illustrate the importance of considering different types of impact when assessing community-based PAR projects in general and in relation to health promotion initiatives engaging communities in EoL issues in particular.
